# Total Preputial Flap: A Reliable and Versatile Technique for Urethral and Penile Reconstruction

**DOI:** 10.3389/fped.2014.00043

**Published:** 2014-05-14

**Authors:** Barbara Ludwikowski, Ricardo González

**Affiliations:** ^1^Pediatric Surgery and Urology, Auf der Bult Kinder- und Jugendkrankenhaus, Hannover, Germany

**Keywords:** hypospadias, preputial flap, onlay urethroplasty, chordee, island flap, epispadias

## Abstract

We revisit the technique of total preputial flap (TPF) and its application for urethroplasty, penile skin coverage of both and present our results in 43 patients (41 hypospadias, 2 epispadias). There were no instances of flap necrosis. In patients without prior attempts at reconstruction (*n* = 36), we observed four urethrocutaneous fistulas. TPF allowed the repair of cases of proximal hypospadias in one stage with an acceptable complication rate.

## Introduction

The repair of hypospadias of moderate to severe grades requires straightening of the penis, augmentation of the existing urethral plate or creation of a neourethra, and replacement of the deficient ventral penile skin. In the first half of the twentieth century, this was usually accomplished in planned staged operations until Hodgson (1) and Asopa *et al*. (2) re-introduced the concept of preputial flaps in the early 1970s. The concept was further developed by Standoli in 1979 (3, 4) and widely popularized by Duckett in 1980 (5). In the last 20 years, many surgeons have tended to return to two-stage repairs following the lead of Greenfield *et al*. (6) and Bracka (7) among others. The reasons for this return to the multistage operations (8) cannot be entirely explained by the results reported in the literature and probably has its origins in the cyclical fashion swings, the apparent simplicity of staged procedures, and the lack of teaching the techniques involved in creating vascularized island flaps in many Pediatric Urology training programs.

Nevertheless, we have continued to use preputial flaps in the manner described in 1996 (9, 10) and perform planned staged procedures only exceptionally. The technique for proximal hypospadias repair, which one of the authors described in 1996 (DOPF) (9), was based on the concept of the total preputial flap (TPF) in order to increase the reliability of the flap. We have used the TPF as a substitute to Byars flaps to cover the ventral aspect of the penis even when the urethroplasty done did not require a preputial flap or no urethroplasty was necessary. In addition, we have used a modification of the TPF technique to cover the dorsal surface of the penis in epispadias repair. In this paper, we revisit the technique and principles of the TPF, describe its applications, and report a new series of cases operated on by the authors (Barbara Ludwikowski) at one institution.

## Materials and Methods

### Surgical technique

All procedures (except in epispadias) are started in the same manner. A traction suture is placed in the glans. A circumferential incision is made 5 mm proximal to the corona, ventrally the incision courses proximal to the urethral meatus and the penile skin is degloved along Buck’s fascia. The correct plane of dorsal dissection is important to avoid bleeding and to preserve the blood supply to the prepuce and dorsal penile skin. Once the mobilization reaches the base of the penis, the correction of chordee and urethroplasty, if other than an onlay is required, are performed. When the ventral dissection of the chordee is not sufficient to achieve a perfectly straight artificial erection, a midline dorsal plication is done using the Heineke–Mikulicz principle after mobilizing the dorsal neurovascular bundles.

**Figure 1:**
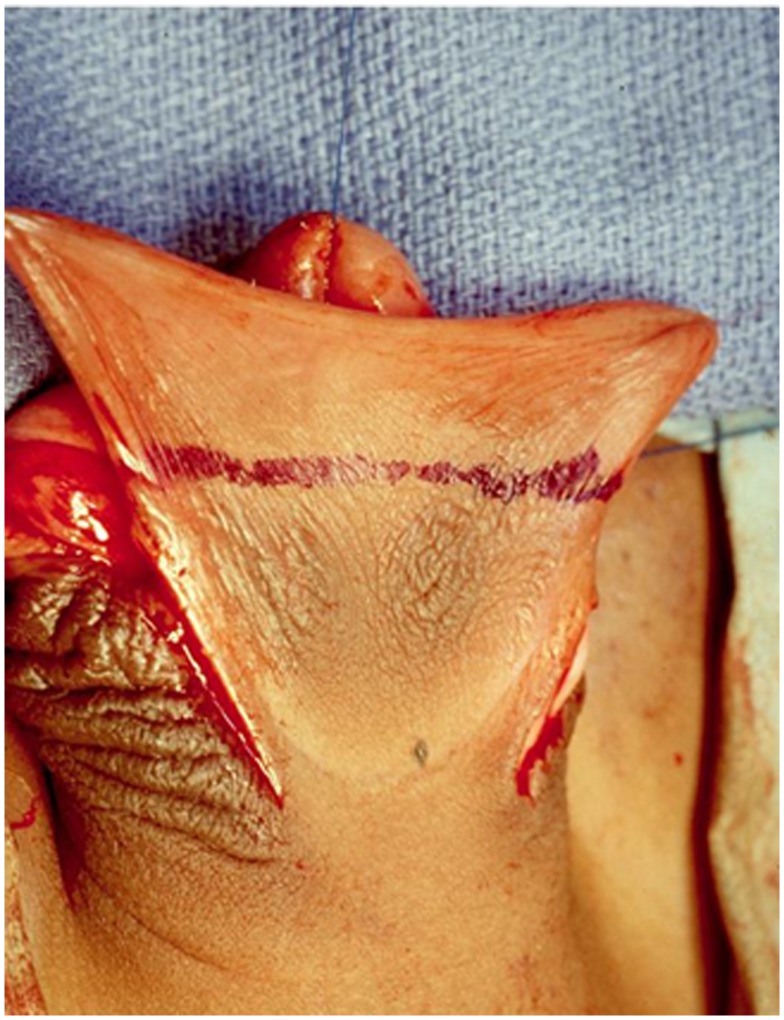
**After the penis is degloved, a superficial incision is made at the junction between the dorsal shaft skin and the prepuce (blue line)**. If there is some degree of scrotal transposition, superficial incisions are made to separate the scrotum from penile shaft. This is important to have enough dorsal skin and to correct the transposition.

**Figure 2:**
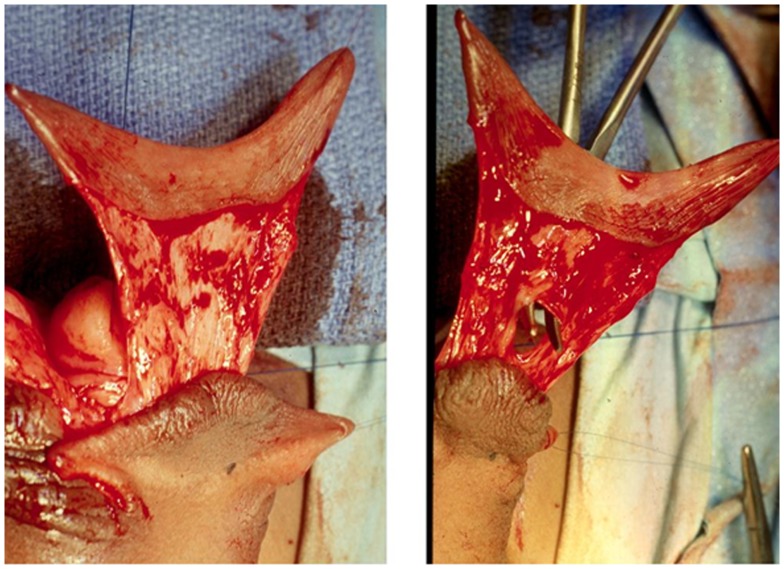
**The left panel shows the dissection of the pedicle for the TPF**. It is important that the dissection plane is close to the dorsal skin. Use of mono or bipolar coagulation should be strictly avoided. The right panel illustrates the creation of a buttonhole at the base of the pedicle used to transpose the flap vertically. The buttonhole is created bluntly and enlarged in a longitudinal direction to avoid vascular injury.

**Figure 3:**
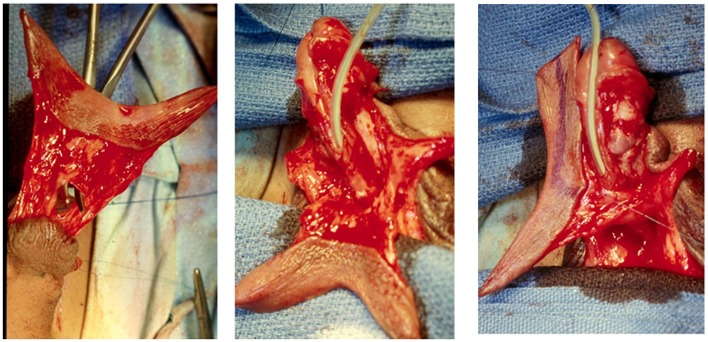
**The TPF has been transposed ventrally and is normally rotated 90°**. This does not compromise the blood supply. It is then used for the onlay urethroplasty (outer layer), ventral coverage, or both. The patient in the illustration had penoscrotal hypospadias. The chordee was corrected with preservation of the urethral plate and the TPF was used for a double face preputial island flap (DOPF). The area marked with blue pencil was used for the onlay and part of the remaining flap for ventral coverage.

Traction sutures are placed at the junction of the prepuce and dorsal shaft skin and a superficial skin incision is made at this point. If the limit between dorsal skin and prepuce is not clear, the location of the sutures is determined by stretching the mobilized dorsal skin and prepuce slightly over the stretched penis. The corona marks the place of the future incision between dorsal skin and prepuce. If there is some degree of penoscrotal transposition, superficial incision are made to separate the dorsal shaft skin from the scrotum (Figure 1). The pedicle of the TPF is prepared by dissecting close to the dorsal penile skin. When the dissection is done following the correct plane, no bleeding should ensue. Hemostasis by mono or bipolar coagulation is strictly avoided at this stage. A buttonhole is created bluntly with a hemostat in the midline at the base of the pedicle and it is gently spread longitudinally (Figure 2). The TPF is then transposed ventral to the shaft (Figure 3) and used for urethroplasty and ventral coverage or both according to the situation.

After obtaining institutional ethical approval, we conducted a retrospective record review of the last 110 hypospadias and 3 epispadias repair operations at our institution over a 3-year period. After surgery all patients were followed by one of the authors (Barbara Ludwikowski). Records of patients whose repair included the use of TPF were reviewed in detail. Data regarding demographics, diagnosis, operation performed, length of follow-up outcome particularly regarding viability of the flap, and complications requiring reoperation were extracted and tabulated. The indications to use a TPF included a proximal hypospadias requiring extensive urethroplasty, lack of sufficient skin to cover the ventral side of the penis after any type of urethroplasty, and most cases of epispadias repair average when needed.

We used the TPF for an onlay urethroplasty when the urethral plate was narrow and could not be reconstructed with either a simple tubularization (Thiersch–Duplay) or meatal based flap (Mathieu).

The TPF was used for ventral coverage when direct approximation of the ventral skin in the midline was not possible without tension. We used the TPF for both the urethroplasty and ventral skin coverage when needed according to the criteria just described.

## Results

A TPF was used in 41 of 110 hypospadias operations (37%) and in 2 of epispadias repairs. The mean age at the time of the operation was 24 months (range 5–137). The TPF was used as part of a double face preputial flap hypospadias repair (DOPF) in 21 cases, to provide tissue for coverage of the ventral surface of the penis only in 15 cases (including dorsal coverage in 2 epispadias), and to provide tissue for an onlay urethroplasty only in 5 cases. Four patients (three DOPF, one onlay urethroplasty only) had previous attempts at hypospadias repair elsewhere. In the same group of 110 consecutive operations, a staged repair was done in five patients (4.7%) three of whom had previous failed repairs done elsewhere.

The mean follow-up period was 11 months (1–42), no patient was lost to follow-up. There were no instances of flap necrosis. Thirty-one patients required simultaneous correction of ventral curvature that persisted after degloving the penile skin. In 21 of these a dorsal plication was necessary to correct the curvature. In only one case, we deemed division of the urethral plate necessary. Of the patients who underwent DOPF, four developed urethrocutaneous fistulas (two were re-operations) for a fistula rate of 10% (2/18) of not previously operated patients. Two children with a small glans, one had a 45X, 46XY karyotype, had a glans dehiscence. All complications were resolved with one additional procedure. One of four patients who had an onlay urethroplasty only developed a small urethrocutaneous fistula. We have not observed any urethral diverticula (Figure 5).

**Figure 4:**
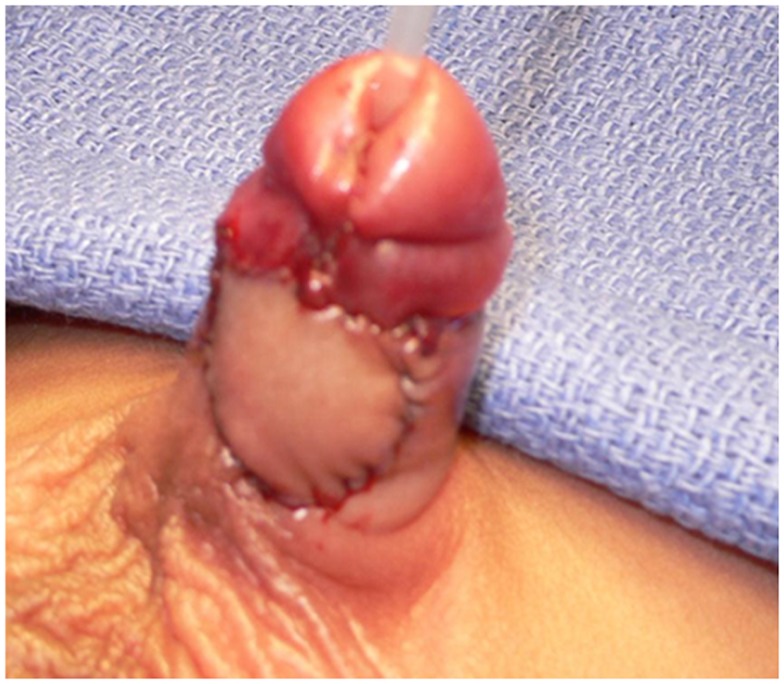
**TPF used for ventral coverage only in patient with severe chordee**. The urethroplasty was done with a meatal based flap.

For patients in whom the TPF was used only to cover the ventral surface of the penis, seven had a simple tubularization of the urethral plate (Thiersch–Duplay), two had a meatal based flap urethroplasty (Figure 4) (modified Mathieu), one a urethral advancement (Beck), and two epispadias repair (Figure 6). No urethral complications were observed in these patients.

**Figure 5:**
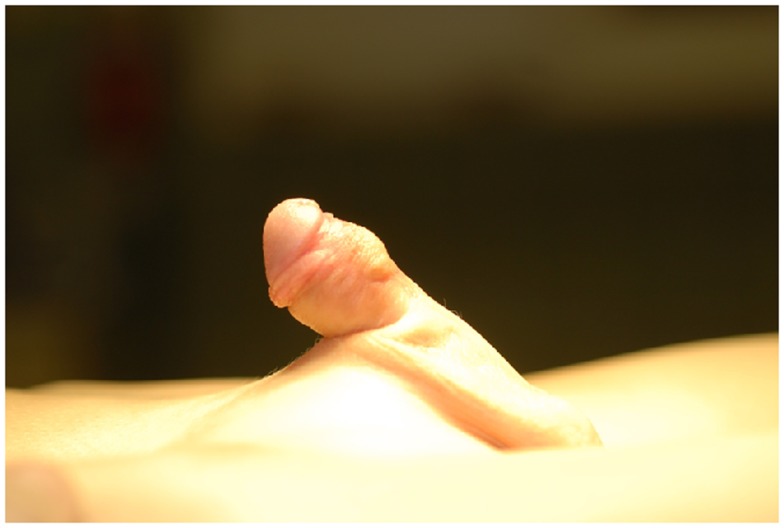
**Appearance of the penis 3 months post DOPF**.

**Figure 6:**
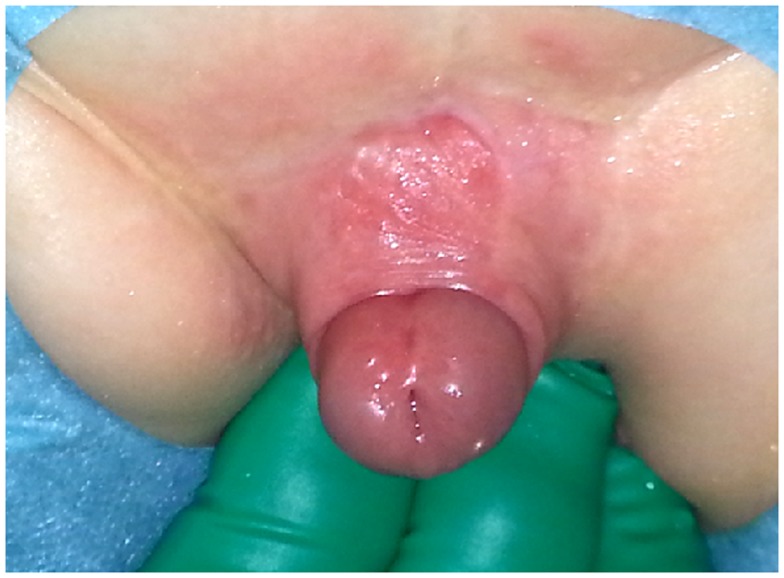
**TPF used for dorsal skin coverage in a case of epispadias (Courtesy Dr. Anja Lingnau, Universitätsmedizin Berlin Charité)**.

## Discussion

The creation of flaps from the dorsal preputial hood and their transfer to the ventral side of the penis have been used for more than a century and are most commonly created by splitting the prepuce in the midline and rotating it to both sides to reach the ventral surface of the penis (11). The Byars technique has been combined with the use of preputial island flaps used for urethral reconstruction (5). The disadvantages of this combination of techniques resides in the fact that the preputial skin blood supply may be compromised in the process of harvesting the pedicle of the island flap leading to partial flap necrosis.

The technique of TPF obviates some of the problems we encountered with the more popular techniques to obtain preputial flaps described by Duckett (5) as mentioned above. We prefer to use the thicker outer prepuce for the urethroplasty to minimize the development of diverticula, which are rather common in some reported series. It should be noted that Standoli also recommended the outer preputial layer for the urethroplasty (4). The technique of bringing the TPF ventrally by means of a buttonhole created at the base of the pedicle avoids axial rotation of the penis that can occur when the flap is transferred ventrally around one side.

In this series and in previous ones we have not experienced flap necrosis in any case. The development of early urethrocutaneous fistulas can be interpreted as evidence of poor blood supply to the flap when the TPF is used for the urethroplasty. A fistula rate of 10% for not previously operated cases is acceptable and similar to that reported previously. However, it is not the intention of this paper to report the results of the urethroplasties since we recognize that much longer follow-up is needed to draw valid conclusions. Instead the purpose of this report is to highlight the reliability of the TPF.

With the systematic preservation of the urethral plate in most cases and the use of the TPF, we have been able to perform one-stage repairs in the vast majority of proximal hypospadias and only rarely resort to planned staged procedures. This group of patients will continue to be followed and the results of the hypospadias repair reported at a later date when sufficient follow-up information is available.

However, others have reported good long-term results of preputial flap urethroplasties (12) which are consistent with our experience.

We reserve the use of a staged procedure for selected case in which the urethral plate needs to be divided, a maneuver we can avoid in most cases, and for re-operative cases with scant penile skin.

The limitations of this study are its retrospective nature, the small number of patients, and the short period of follow-up. However, since the purpose of the report is to evaluate the outcome of the TPF and flap necrosis occurs immediately after surgery, we believe 11 months is a sufficient time to evaluate the outcome of the flap.

## Conclusion

Total preputial flap is a reliable and versatile technique to create a preputial flap that has allowed the authors to repair most cases of proximal hypospadias in one stage. This technique avoids the use of Byars flaps and is also useful in epispadias repair.

## Conflict of Interest Statement

The authors declare that the research was conducted in the absence of any commercial or financial relationships that could be construed as a potential conflict of interest.
